# Krasnoselskii-type algorithm for zeros of strongly monotone Lipschitz maps in classical banach spaces

**DOI:** 10.1186/s40064-015-1044-1

**Published:** 2015-06-27

**Authors:** C E Chidume, A U Bello, B Usman

**Affiliations:** African University of Science and Technology, Abuja, Nigeria; Federal University, Dutsin-Ma, Dutsin-Ma, Katsina State Nigeria

**Keywords:** Strongly monotone, Lipschitz, Hölder continiuty

## Abstract

Let $E=L_p$, $1<p<\infty$, and $A:E\rightarrow E^*$ be a strongly monotone and Lipschitz mapping. A Krasnoselskii-type sequence is constructed and proved to converge strongly to the unique solution of $Au=0$. Furthermore, our technique of proo f is of independent interest.

## Background

Let $H$ be a real Hilbert space. An operator $A:H\rightarrow H$ is called *monotone* if

1.1 $$\begin{aligned} \big <Ax-Ay,x-y\big >\ge 0\quad\forall ~x,y\in H, \end{aligned}$$

and is called *strongly monotone* if there exists $\lambda \in (0,1)$ such that

1.2 $$\begin{aligned} \big <Ax-Ay,x-y\big >\ge \lambda \Vert x-y\Vert ^2 \quad\forall x,y\in H. \end{aligned}$$

Interest in monotone operators stems mainly from their usefulness in numerous applications. Consider, for example, the following: Let $f:H\rightarrow \mathbb {R}$ be a proper and convex function. The *subdifferential* of $f$ at $x\in H$ is defined by

$$\begin{aligned} \partial f(x)=\big \{x^*\in H:f(y)-f(x)\ge \big <y-x,x^*\big >\quad\forall ~y\in H\big \}. \end{aligned}$$

It is easy to check that $\partial f:H\rightarrow 2^H$ is a *monotone operator* on $H$, and that $0\in \partial f(x)$ if and only if $x$ is a minimizer of $f$. Setting $\partial f\equiv A$, it follows that solving the inclusion $0\in Au$, in this case, is solving for a minimizer of $f$.

Let $E$ be a real normed space, $E^*$ its topological dual space. The map $J:E\rightarrow 2^{E^*}$ defined by

$$\begin{aligned} Jx=\big \{x^*\in E^*:\big <x,x^*\big >=\Vert x\Vert .\Vert x^*\Vert ,~\Vert x\Vert =\Vert x^*\Vert \big \} \end{aligned}$$

is called the *normalized duality map* on $E$. A map $A:E\rightarrow E$ is called *accretive* if for each $x,y\in E$, there exists $j(x-y)\in J(x-y)$ such that

1.3 $$\begin{aligned} \big <Ax-Ay,j(x-y)\big >\ge 0. \end{aligned}$$

$A$ is called *strongly accretive * if there exists $k\in (0,1)$ such that for each $x,y\in E$, there exists $j(x-y)\in J(x-y)$ such that

1.4 $$\begin{aligned} \big <Ax-Ay,j(x-y)\big > \ge k\Vert x-y\Vert ^2. \end{aligned}$$

Several existence theorems have been established for the equation $Au=0$ when $A$ is of the monotone-type (see e.g., Deimling (1985; Pascali and Sburian 1978).

For approximating a solution of $Au=0$, assuming existence, where $A:E\rightarrow E$ is of accretive-type, Browder (1967) defined an operator $T:E \rightarrow E$ by $T:=I-A$, where $I$ is the identity map on $E$. He called such an operator *pseudo-contractive*. A map $T:E\rightarrow E$ is then called *pseudo-contractive* if

1.5 $$\begin{aligned} \big <Tx-Ty,x-y\big > \le \Vert x-y\Vert ^2\quad \forall x,y \in E, \end{aligned}$$

and is called *strongly pseudo-contractive* if there exists $k\in (0,1)$ such that

1.6 $$\begin{aligned} \big <Tx-Ty,x-y\big > \le k\Vert x-y\Vert ^2\quad \forall x,y \in E. \end{aligned}$$

It is trivial to observe that zeros of $A$ corresspond to fixed points of $T$. For Lipschitz strongly pseudo-contractive maps, Chidume (1987) proved the following theorem.

### **Theorem C1**

(Chidume 1987)* Let*$E =L_{p}, ~2\le p < \infty$*, and*$K\subset E$* be nonempty closed convex and bounded. Let*$T:K\rightarrow K$* be a strongly pseudocontractive and Lipschitz map. For arbitrary*$x_{0}\in K$*, let a sequence*$\{x_{n}\}$*be defined iteratively by*$x_{n+1} = (1-\alpha _{n})x_{n} + \alpha _{n}Tx_{n},~n\ge 0,$*where*$\{\alpha _{n}\} \subset (0,1)$*satisfies the following conditions:*$(i)~\sum _{n=1}^{\infty }\alpha _{n}= \infty , ~~(ii)\sum _{n=1}^{\infty }\alpha _{n}^{2} < \infty$*. Then,*$\{x_{n}\}$*converges strongly to the unique fixed point of*$T$.

The main tool used in the proof of Theorem C1 is an inequality of Bynum (1976). This theorem signalled the return to extensive research efforts on inequalities in Banach spaces and their applications to iterative methods for solutions of nonlinear equations. Consequently, this theorem of Chidume has been generalized and extended in various directions, leading to flourishing areas of research, for the past thirty years or so, by numerous authors (see e.g., Chidume 1986, 1990, 2002; Chidume and Ali 2007; Chidume and Chidume 2005, 2006; Chidume and Osilike 1999; Deng 1993a, b; Zhou 1997; Zhou and Jia 1996, 1997; Liu 1995, 1997; Qihou 1990, 2002; Weng 1991, 1992; Xiao 1998; Xu 1989, 1991a, b, 1992, 1998; Xu and Roach 1991, 1992; Xu et al. 1995; Zhu 1994 and a host of other authors). Recent monographs emanating from these researches include those by Chidume (2009), Berinde (2007), Goebel and Reich (1984) and William and Shahzad (2014).

Unfortunately, the success achieved in using geometric properties developed from the mid 1980ies to early 1990ies in approximating zeros of accretive-type mappings has not carried over to approximating zeros of monotone-type operators in general Banach spaces. The first problem is that since $A$ maps $E$ to $E^{*}$, for $x_{n}\in E$, $Ax_{n}$ is in $E^{*}$. Consequently, a recursion formula containing $x_{n}$ and $Ax_{n}$ may not be well defined. Another difficulty is that the normalized duality map which appears in most Banach space inequalities developed, and also appears in the definition of accretive-type mappings, does not appear in the definition of monotone-type mappings in general Banach spaces. This creats very serious technical difficulties.

Attemps have been made to overcome the first difficulty by introducing the inverse of the normalized duality mapping in the recursion formulas for approximating zeros of monotone-type mappings. But one major problem with such recursion formulas is that the exact form of the normalized duality map (or its inverse) is not known precisely in any space more general than $L_p$ spaces, $1<p<\infty$. Futhermore, the recursion formulas, apart from containing the normalized duality map and its inverse, generally involve computation of subsets and generalized projections, both of which are defined in a way that makes their computation almost impossible. We give some examples of some results obtained using these approximation schemes. Before we do this, however, we need the following definitions.

Let $E$ be a real normed space and let a funtion $\phi (.,.):X\times X\longrightarrow \mathbb {R}$ be defined by

$$\begin{aligned} \phi (x,y)= ||x\Vert ^2-2\big <x,J(y)\big > +||y||^2 \quad\forall ~x, y \in E. \end{aligned}$$

It is easy to see that in Hilbert space, $\phi (x,y)$ reduces to $\Vert x-y\Vert ^2$. A function $\pi _K:E\longrightarrow K$ defined by: $\pi _K(x)=\bar{x}$ such that $\bar{x}$ is the solution of

$$\begin{aligned} \min \big \{\phi (x,y),y\in K \big \}, \end{aligned}$$

is called a *generalized projection map.*

Now we present the following results.

In Hilbert space, suppose that a map $A:K\rightarrow H$ is $\gamma$-inverse strongly monotone, i.e., there exists $\gamma >0$ such that $\langle Ax-Ay, x-y\rangle \ge \gamma ||Ax-Ay||^{2} ~\forall ~x, y\in H$. Iiduka et al. (2004) studied the following iterative scheme.

1.7 $$\begin{aligned} \left\{ \begin{array}{lll} x_0&\in &K, choosen ~~arbitrary, \\ y_n&=&P_K(x_n-\alpha _nAx_n);\\ C_n&=&\big \{z\in K: \Vert y_n-z\Vert \le \Vert x_n-z\Vert \big \},\\ Q_n&=&\big \{z\in K:\big <x_n-z,x_0-x_n\big >\ge 0\big \}\\ x_{n+1}&=&P_{C_n\cap Q_n} (x_0), n\ge 1, \end{array} \right. \end{aligned}$$

where $\{\alpha _n \}$ is a sequence in $[0, 2\gamma ]$. They proved that the sequence $\{x_n \}$ generated by (1.7) converges strongly to $P_{VI(K ,A)} (x_0 )$, where $P_{VI(K ,A)}$ is the metric projection from $K$ onto $VI(K , A)$ (see e.g., Iiduka et al. 2004 for definition and explanation of the symbols).

Zegeye and Shahzad proved the following result.

### **Theorem 1.1**

(Zegeye and Shahzad 2009)* Let*$E$* be uniformly smooth and*$2$*-uniformly convex real Banach space with dual*$E^*$*. Let*

$A:E\longrightarrow E^*$*be a *$\gamma$*-inverse strongly monotone mapping and*

$T:E\longrightarrow E$*be relatively weak nonexpansive mapping with*$A^{-1}(0)\cap F(T)\ne \emptyset .$*Assume that*$0<\alpha _n\le b_0:=\frac{\gamma c^2}{2},$*where*$c$*is the constants from the Lipschitz property of*$J^{-1}$*, then the sequence generated by*

$$\begin{aligned} \left\{ \begin{array}{lll} x_0&\in&K, choosen ~~arbitrary, \\ y_n&=&J^{-1}(Jx_n-\alpha _nAx_n);\\ z_n&=&Ty_n,\\ H_0&=&\big \{v\in K:\phi (v,z_0)\le \phi (v,y_0)\le \phi (v,x_0)\big \},\\ H_n&=&\big \{v\in H_{n-1}\cap W_{n-1}:\phi (v,z_n)\le \phi (v,y_n)\le \phi (v,x_n)\big \},\\ W_0&=&E,\\ W_n&=&\big \{v\in W_{n-1}\cap H_{n-1}:\big <x_n-v,jx_0-jx_n\big >\ge 0\big \}\\ x_{n+1}&=&\Pi _{H_n\cap W_n} (x_0), n\ge 1, \end{array} \right. \end{aligned}$$

*converges strongly to*$\Pi _{F(T)\cap A^{-1}(0)}x_0$*where*$\Pi _{F(T)\cap A^{-1}(0)}$*is the generalised projection from*$E$*onto*$F(T)\cap A^{-1}(0).$

We remark here that although the approximation methods used in the result of Iiduka *et al.* referred to above, and in Theorem 1.1 yield strong convergence to a solution of the problem under consideration, it is clear that they are not easy to implement. Furthermore, Theorem 1.1 excludes $L_p$ spaces, $2<p<\infty$, because these spaces are *not*$2$-uniformly convex. The theorem, however, is applicable in $L_{p}$ spaces $1<p<2$.

In this paper, we introduce an *iterative scheme of Krasnoselskii-type* to approximate the unique zero of *a strongly monotone Lipschitz mapping* in $L_{p}$ spaces, $1<p<\infty$. In these spaces, the formula for $J$ is known precisely (see e.g., Cioranescu 1990; Chidume 2009). The Krasnoselskii sequence, whenever it converges, is known to converge as fast as a geometric progression. Furthermore, our iteration method which will not involve construction of subsets or the use of generalized projection is also of independent interest.

## Preliminaries

In the sequel, we shall need the following results and definitions.

### **Lemma 2.1**

(see e.g., Chidume 2009, p. 55)* Let*$E=L_{p},\,1<p<2.$* Then, there exists a constant*$c_{p}>0$*such that for all x, y in L*_*p*_*the following inequalities hold:*

2.1 $$\begin{aligned} \Vert x+y\Vert ^2\ge & {} \Vert x\Vert ^2+2\langle y,J(x)\rangle +c_p\Vert y\Vert ^2, \end{aligned}$$

2.2 $$\begin{aligned} \langle x-y,J(x)-J(y)\rangle \ge (p-1)\Vert x-y\Vert ^2. \end{aligned}$$

Let $E$ be a smooth real Banach space with dual $E^*$. The function $\phi :E\times E\rightarrow \mathbb {R}$, defined by,

2.3 $$\begin{aligned} \phi (x,y)=\Vert x\Vert ^2-2\langle x,Jy\rangle +\Vert y\Vert ^2 \quad \text {for}~x,y\in E, \end{aligned}$$

where $J$ is the normalized duality mapping from $E$ into $2^{E^*}$, introduced by Alber has been studied by Alber (1996), Alber and Guerre-Delabriere (2001), Kamimura and Takahashi (2002), Reich (1996) and a host of other authors. If $E=H$, a real Hilbert space, then Eq (2.3) reduces to $\phi (x,y)=\Vert x-y\Vert ^2$ for $x,y\in H.$ It is obvious from the definition of the function $\phi$ that

2.4 $$\begin{aligned} (\Vert x\Vert -\Vert y\Vert )^2\le \phi (x,y)\le (\Vert x\Vert +\Vert y\Vert )^2\quad\text {for}~x,y\in E. \end{aligned}$$

Define $$V:X\times X^*\rightarrow \mathbb {R}$$ by

2.5 $$\begin{aligned} V(x,x^*)=\Vert x\Vert ^2-2\langle x,x^*\rangle +\Vert x^*\Vert ^2. \end{aligned}$$

Then, it is easy to see that

2.6 $$\begin{aligned} V(x,x^*)=\phi (x,J^{-1}(x^*)) \quad\forall ~x\in ~X,~x^*\in ~X^*. \end{aligned}$$

### **Corollary 2.2**

*Let *$E=L_p$, $1<p\le 2$*. Then *$J^{-1}$*is Lipschitz, i.e., there exists*$L_1>0$*such that for all*$u,v\in E^*$*, the following inequality holds:*

2.7 $$\begin{aligned} \Vert J^{-1}(u)-J^{-1}(v)\Vert \le L_1\Vert u-v\Vert . \end{aligned}$$

### *Proof*

This follows from inequality (2.2). $\square$

For $L_p,~2\le p<\infty$, we have the following lemma.

### **Lemma 2.3**

(Alber and Ryazantseva 2006, p. 48)* Let*$X=L_p,~p\ge 2$*. Then, the inverse of the normalized duality map*$J^{-1}:X^*\rightarrow X$*is Hölder continuous on balls. i.e.,*$\forall ~ u,v\in X^*$*such that*$\Vert u\Vert \le R$*and*$\Vert v\Vert \le R$*, then*

2.8 $$\begin{aligned} \Vert J^{-1}(u)-J^{-1}(v)\Vert \le m_p\Vert u-v\Vert ^\frac{1}{p-1}, \end{aligned}$$

*where*$m_p:=(2^{p+1}Lpc^p_2)^{\frac{1}{p-1}}>0$*, for some constant*$c_2>0.$

### *Proof*

This follows from the following inequality:

2.9 $$\begin{aligned} \langle Jx-Jy,x-y\rangle \ge \frac{\Vert x-y\Vert ^p}{2^{p+1}Lpc^p_2},~~~c_2=2\max \{1,R\}. \end{aligned}$$

(see e.g., Alber and Ryanzantseva 2006, p. 48). $\square$

### **Lemma 2.4**

(Alber 1996)* Let*$X$* be a reflexive striclty convex and smooth Banach space with*$X^*$* as its dual. Then,*

2.10 $$\begin{aligned} V(x,x^*)+2\langle J^{-1}x^*-x,y^*\rangle \le V(x,x^*+y^*) \end{aligned}$$

*for all*$x\in X$*and*$x^*,y^*\in X^{*}.$

### **Definition 2.5**

An operator $T:X\rightarrow X^*$ is called $\psi$-*strongly monotone* if there exists a continuous, strictly increasing function $\psi :\mathbb {R}\rightarrow \mathbb {R}$ with $\psi (0)=0$ such that

2.11 $$\begin{aligned} \big <Tx-Ty,x-y\big >\ge \psi (\Vert x-y\Vert )\Vert x-y\Vert \quad \forall ~x,y\in D(T). \end{aligned}$$

Let $X$ and $Y$ be Banach spaces with $X^*$ and $Y^*$ as their respective duals.

### **Definition 2.6**

An operator $A:D(A)\subset X\rightarrow Y^*$ is called *hemicontinuous* at $x_0\in D(A)$ if $x_0+t_ny\in D(A)$,

$$\begin{aligned} for ~y\in X~and~t_n\rightarrow 0_{+}~ \implies ~A(x_0+t_ny)\mathop {\rightarrow }\limits ^{w^*} Ax_0. \end{aligned}$$

Clearly, every continuous map is hemicontinuous.

### **Lemma 2.7**

*Let*$T:X\rightarrow X^*$* be a hemicontinuou*s $\psi$*-strongly monotone operator with*$D(T)=X$*. Then,*$R(T)=X^*.$

### *Proof*

See chapter III, page $48$ of Pascali and Sburian (1978). $\square$

## Main results

## Convergence in $L_p$ spaces, $1<p\le 2$.

In the sequel, $k$ is the strong monotonicity constant of $A$ and $L>0$ is its Lipschitz constant, and $\delta :=\frac{k}{2(L_1+1)(L+1)^2}$.

### **Theorem 4.1**

*Let*$E=L_p,~1<p\le 2$*. Let*$A:E\rightarrow E^{*}$* be a strongly monotone and Lipschitz map. For*$x_0\in E$* arbitrary, let the sequence*$\{x_n\}$* be defined by:*

4.1 $$\begin{aligned} x_{n+1} = J^{-1}(Jx_n - \lambda Ax_n), \quad n\ge 0, \end{aligned}$$

*where*$\lambda \in \Big (0,\delta \Big )$*. Then, the sequence*$\{x_n\}$*converges strongly to*$x^*\in A^{-1}(0)$*and*$x^*$* is unique.*

### *Proof*

Let $\psi (t)=kt$ in inequality (2.11). By Lemma 2.7, $A^{-1}(0)\ne \emptyset$. Let $x^*\in A^{-1}(0)$. Using the definition of $x_{n+1}$ we compute as follows:

$$\begin{aligned} \phi (x^*,x_{n+1})&=\phi (x^*,J^{-1}(Jx_n-\lambda Ax_n))\\&= V(x^*,Jx_n-\lambda Ax_n) \end{aligned}$$

Applying Lemma 2.4, we have

$$\begin{aligned} \phi (x^*,x_{n+1})&=V(x^*,Jx_n-\lambda Ax_n)\\&\le V(x^*,Jx_n)-2\lambda \langle J^{-1}(Jx_n-\lambda Ax_n)-x^*,Ax_n-Ax^*\rangle \\&= \phi (x^*,x_n)-2\lambda \langle x_n-x^*,Ax_n-Ax^*\rangle \\&\quad+  2\lambda \langle x_n-x^*,Ax_n-Ax^*\rangle \\& \quad- 2\lambda \langle J^{-1}(Jx_n-\lambda Ax_n)-x^*,Ax_n-Ax^*\rangle \\&= \phi (x^*,x_n)-2\lambda \langle x_n-x^*,Ax_n-Ax^*\rangle \\&\quad - 2\lambda \langle J^{-1}(Jx_n-\lambda Ax_n)-J^{-1}(Jx_n),Ax_n-Ax^*\rangle . \end{aligned}$$

Using the strong monotonocity of $A$, Lipschitz property of $j^{-1}$ and the Lipschitz property of $A$, we have that:

$$\begin{aligned} \phi (x^*,x_{n+1})&\le \phi (x^*,x_n)-2\lambda k\Vert x_n-x^*\Vert ^2\\&\quad+ 2\lambda \Vert J^{-1}(Jx_n-\lambda Ax_n)-J^{-1}(Jx_n)\Vert \Vert Ax_n-Ax^*\Vert \\&\le \phi (x^*,x_n)-2\lambda k\big \Vert x_n-x^*\big \Vert ^2 +2\lambda ^2L_1L^2\big \Vert x_n-x^*\big \Vert ^2\\&\le \phi (x^*,x_n)-\lambda k\Vert x_n-x^*\Vert ^2. \end{aligned}$$

Thus, $\phi (x^*,x_n)$ converges, since it is monotone decreasing and bounded below by zero. Consequently,

$$\begin{aligned} \lambda k\Vert x_n-x^*\Vert ^2\le \phi (x^*,x_n)-\phi (x^*,x_{n+1})\rightarrow 0, \quad as~n\rightarrow \infty . \end{aligned}$$

This yields $x_n\rightarrow x^*~~as~~ n\rightarrow \infty .$ Suppose there exists $y^*\in A^{-1}(0)$, $y^*\ne x^*$. Then, substituting $x^*$ by $y^*$ in the above argument, we obtain that $x_n\rightarrow y^*$ as $n\rightarrow \infty$. By uniqueness of limit $x^*=y^*$. So, $x^*$ is unique. completing the proof. $\square$

## Convergence in $L_p$ spaces, $2\le p<\infty$.

### *Remark 1*

We remark that for $E=L_p,~2\le p<\infty$, if $A:E\rightarrow E^*$ satisfies the following conditions: there exists $k\in (0,1)$ such that

5.1 $$\begin{aligned} \big <Ax-Ay,x-y\big >\ge k\Vert x-y\Vert ^{\frac{p}{p-1}} \quad \forall ~ x,y\in E, \end{aligned}$$

and $A^{-1}(0)\ne \emptyset$, then the *Krasnoselskii-type sequence* (4.1) converges strongly to the unique solution of $Au=0$. In fact, we prove the following theorem.

In the following theorem, $\delta _p:=\Big (\frac{k}{2m_pL^{\frac{p}{p-1}}}\Big )^{p-1}$.

### **Theorem 5.1**

*Let*$X=L_p,~2\le p<\infty$*. Let*$A:X\rightarrow X^*$*be a Lipschitz map. Assume that there exists a constant *$k\in (0,1)$*such that*$A$*satisfies the following condition:*

5.2 $$\begin{aligned} \big <Ax-Ay,x-y\big >\ge k\Vert x-y\Vert ^{\frac{p}{p-1}}, \end{aligned}$$

*and that*$A^{-1}(0)\ne \emptyset .$*For arbitrary*$x_0\in X$*, define the sequence*$\{x_n\}$*iteratively by:*

5.3 $$\begin{aligned} x_{n+1}=J^{-1}(Jx_n-\lambda Ax_n), \quad n\ge 0, \end{aligned}$$

*where*$\lambda \in (0,\delta _p)$*. Then, the sequence*$\{x_n\}$*converges strongly to the unique solution of the equation*$Ax=0.$

### *Proof*

We first prove that $\{x_n\}$ is bounded. This proof is by induction.

Let $x^*\in A^{-1}(0)$. Then, there exists $r>0$ such that $\phi (x^*,x_1)\le r$. By construction, $\phi (x^*,x_1)\le r$. Suppose that $\phi (x^*,x_n)\le r$, for some $n\ge 1$. We prove that $\phi (x^*,x_{n+1})\le r$.

Using Eq (2.6) and inequality (2.10), we have:

$$\begin{aligned} \phi (x^*,x_{n+1})&= \phi (x^*,J^{-1}(Jx_n-\lambda Ax_n)) = V(x^*,Jx_n-\lambda Ax_n)\\&\le V(x^*,Jx_n)-2\langle J^{-1}(Jx_n-\lambda Ax_n)-x^*,\lambda Ax_n\rangle \\&= V(x^*,Jx_n)-2\lambda \langle x_n-x^*,Ax_n-Ax^*\rangle \\&\quad +2\lambda \langle J^{-1}(Jx_n-\lambda Ax_n)-J^{-1}(Jx_n),Ax_n-Ax^*\rangle .\\&\le \phi (x^*,x_n)-2\lambda \langle x_n-x^*,Ax_n-Ax^*\rangle \\& \quad +2\lambda \Vert J^{-1}(Jx_n-\lambda Ax_n)-J^{-1}(Jx_n)\Vert \Vert Ax_n-Ax^*\Vert . \end{aligned}$$

Using condition (5.2) on $A$ and inequality (2.8), we obtain:

$$\begin{aligned} \phi (x^*,x_{n+1})&\le\phi (x^*,x_n)-2k\lambda \Vert x_n-x^*\Vert ^{\frac{p}{p-1}}+2\lambda \lambda ^{\frac{1}{p-1}}m_p\Vert Ax_n\Vert ^{\frac{1}{p-1}}\Vert Ax_n-Ax^*\Vert \\&\le \phi (x^*,x_n)-2k\lambda \Vert x_n-x^*\Vert ^{\frac{p}{p-1}}+2\lambda \lambda ^{\frac{1}{p-1}}m_p\Vert Ax_n-Ax^*\Vert ^{\frac{p}{p-1}}.\\&\le\phi (x^*,x_n)-2k\lambda \Vert x_n-x^*\Vert ^{\frac{p}{p-1}}+2\lambda \lambda ^{\frac{1}{p-1}}m_pL^{\frac{p}{p-1}}\Vert x_n-x^*\Vert ^{\frac{p}{p-1}}\\&\le \phi (x^*,x_n)-k\lambda \Vert x_n-x^*\Vert ^{\frac{p}{p-1}}\\&\le r. \end{aligned}$$

Hence, by induction, $\{x_n\}$ is bounded. We now prove that $\{x_n\}$ converges strongly to $x^*\in A^{-1}(0)$. Let $x^*\in A^{-1}(0)$. From the same computation as above, we have that:

$$\begin{aligned} \phi (x^*,x_{n+1})\le & {} \phi (x^*,x_n)-\lambda k\Vert x_n-x^*\Vert ^{\frac{p}{p-1}}, \end{aligned}$$

which implies $\phi (x^*,x_n)$ is decreasing and bounded below by zero, so the limit of $\phi (x^*,x_n)$ exists. Therefore,

$$\begin{aligned} 0\le \lim \Big (\lambda k\Vert x_n-x^*\Vert ^{\frac{p}{p-1}}\Big )\le \lim \Big (\phi (x^*,x_n)-\phi (x^*,x_{n+1})\Big )=0. \end{aligned}$$

Hence, $x_n\rightarrow x^*$ as $n\rightarrow \infty$. Uniqueness follows as in the proof of Theorem 4.1. $\square$

## Open Question

 If $E=L_p,~2\le p<\infty$, attempts to obtain strong convergence of the *Krasnoselskii-type sequence* defined for $x_0\in E$, by:

5.4 $$\begin{aligned} x_{n+1}=J^{-1}(J(x_n)-\lambda Ax_n), \quad n\ge 0, \lambda \in (0,1) \end{aligned}$$

to a solution of the equation $Au=0$, where $A$ is strongly monotone and Lipschitz, have not yielded any positive result. It is, therefore, of interest to find out if a *Krasnoselskii-type sequence* will converge strongly to a solution of $Au=0$*in this space*.
